# Point-of-care Ultrasound Diagnosis of Type B Aortic Dissection on the Suprasternal Notch View

**DOI:** 10.7759/cureus.6005

**Published:** 2019-10-27

**Authors:** Richard N Wang, Michelle Escobar, Shamicka North, Judy Lin

**Affiliations:** 1 Emergency Medicine, Maimonides Medical Center, Brooklyn, USA; 2 Pediatric Emergency Medicine, Maimonides Medical Center, Brooklyn, USA

**Keywords:** aortic dissection, emergency ultrasound, point of care ultrasound, suprasternal

## Abstract

Aortic dissection (AD) is a life-threatening but uncommon and challenging diagnosis to make in the emergency department. Cardiac point-of-care ultrasound (POCUS) is often used to evaluate patients with chest pain and may be used to rapidly diagnose Stanford type A AD on the suprasternal notch view if there is the visualization of a dissection flap. In contrast, a diagnosis of type B AD on the suprasternal notch view is rare and has only been reported in one previous case report. We report the case of a patient who presented with chest pain and was accurately diagnosed with type B AD using the suprasternal notch view.

## Introduction

Aortic dissection (AD) is a life-threatening but uncommon and challenging diagnosis to make in the emergency department (ED). While a sudden onset of severe pain is the most common presenting complaint, it can have a wide range of manifestations and classic findings are often absent [1]. Computed tomography angiography (CTA) is the most common initial assessment performed and has high sensitivity and specificity [1-2]. The Stanford classification divides AD into type A, which involves the ascending aorta, and type B, which is limited to the aorta distal to the left subclavian artery. Cardiac point-of-care ultrasound (POCUS) is often used to evaluate patients with chest pain and may be used to rapidly diagnose type A AD in the suprasternal notch view if there is the visualization of a dissection flap [3]. In contrast, a diagnosis of type B AD in the suprasternal notch view is rare and has only been reported in one previous case report. We report the case of a patient who presented with chest pain and was accurately diagnosed with type B AD using the suprasternal notch view.

## Case presentation

A 77-year-old male with a history of hypertension presented to the ED with the chief complaint of chest pain. He described excruciating substernal chest pain that was sudden onset, non-radiating, non-pleuritic, and without associated nausea, vomiting, diaphoresis, or shortness of breath.

**Figure 1:**
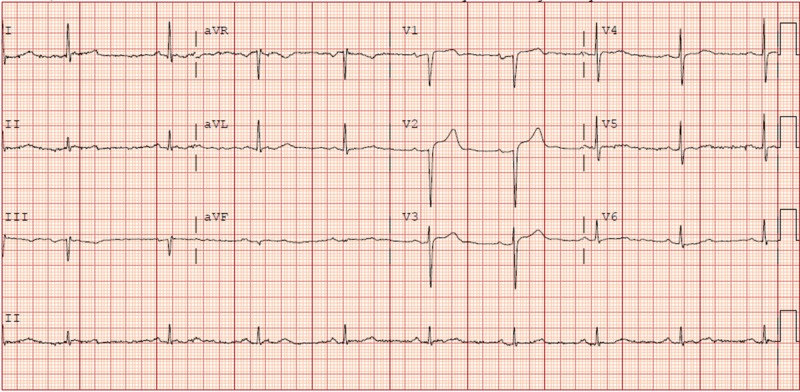
ECG ECG: electrocardiogram

**Figure 2:**
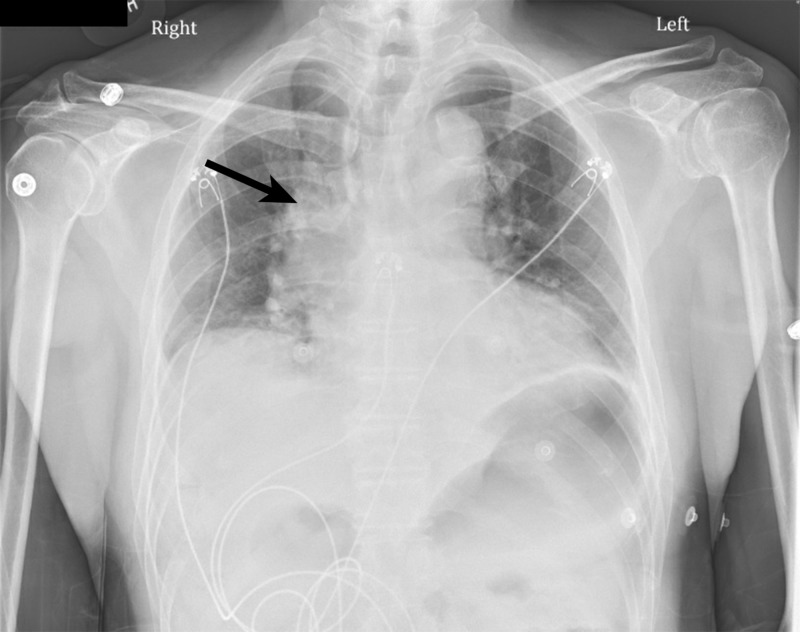
CXR with fullness of the right hilum CXR: chest X-ray

His initial vitals were as follows: temperature 97.7°F, heart rate 57 beats/minute, respiratory rate 18 breaths/min, blood pressure 131/64 mmHg, and oxygen saturation 95% on room air. His physical exam was unremarkable. His laboratory results were notable for a white blood cell (WBC) count of 15,100 cells per microliter, normal basic metabolic panel, and two sets of cardiac troponin I negative. An electrocardiogram (ECG) showed ST-elevation in V2 and V3 without reciprocal changes (Figure 1). Chest X-ray showed bibasal atelectasis and fullness of the right hilum (Figure 2).

**Figure 3:**
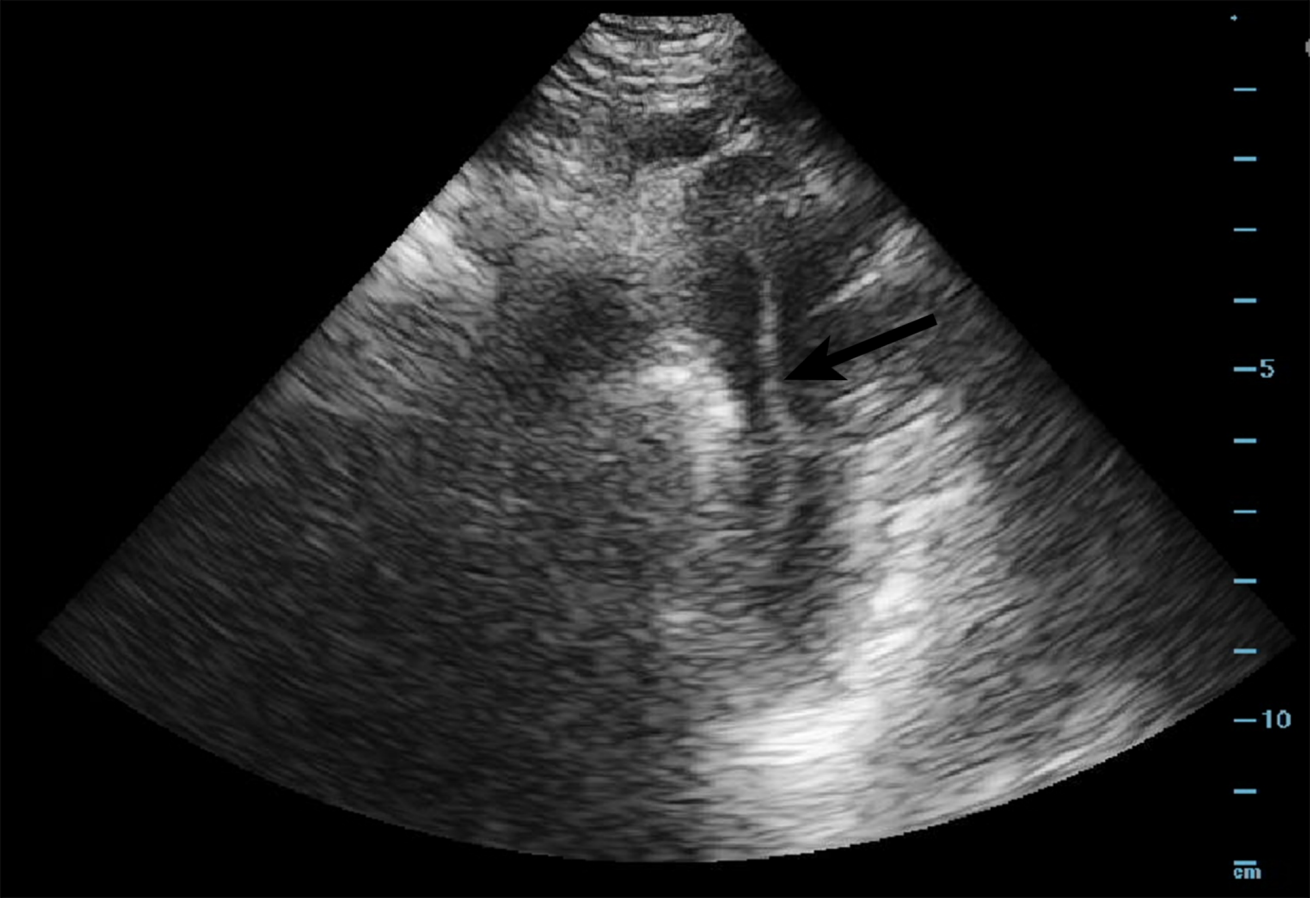
Suprasternal notch view with visualization of a dissection flap

About six hours after initial presentation, the patient was noted to be hypertensive to 200/95 mmHg with concern of pain moving to the middle of the abdomen. POCUS of the abdominal aorta at that time revealed an area concerning for an intimal flap, which was more clearly appreciated on the cardiac suprasternal notch view (Videos 1-3, Figure 3).

**Video 1:** Abdominal aorta (transverse view) with suggestion of dissection flap

**Video 2:** Abdominal aorta (longitudinal view)

**Video 3:** Suprasternal notch view with clear visualization of dissection flap

**Figure 4:**
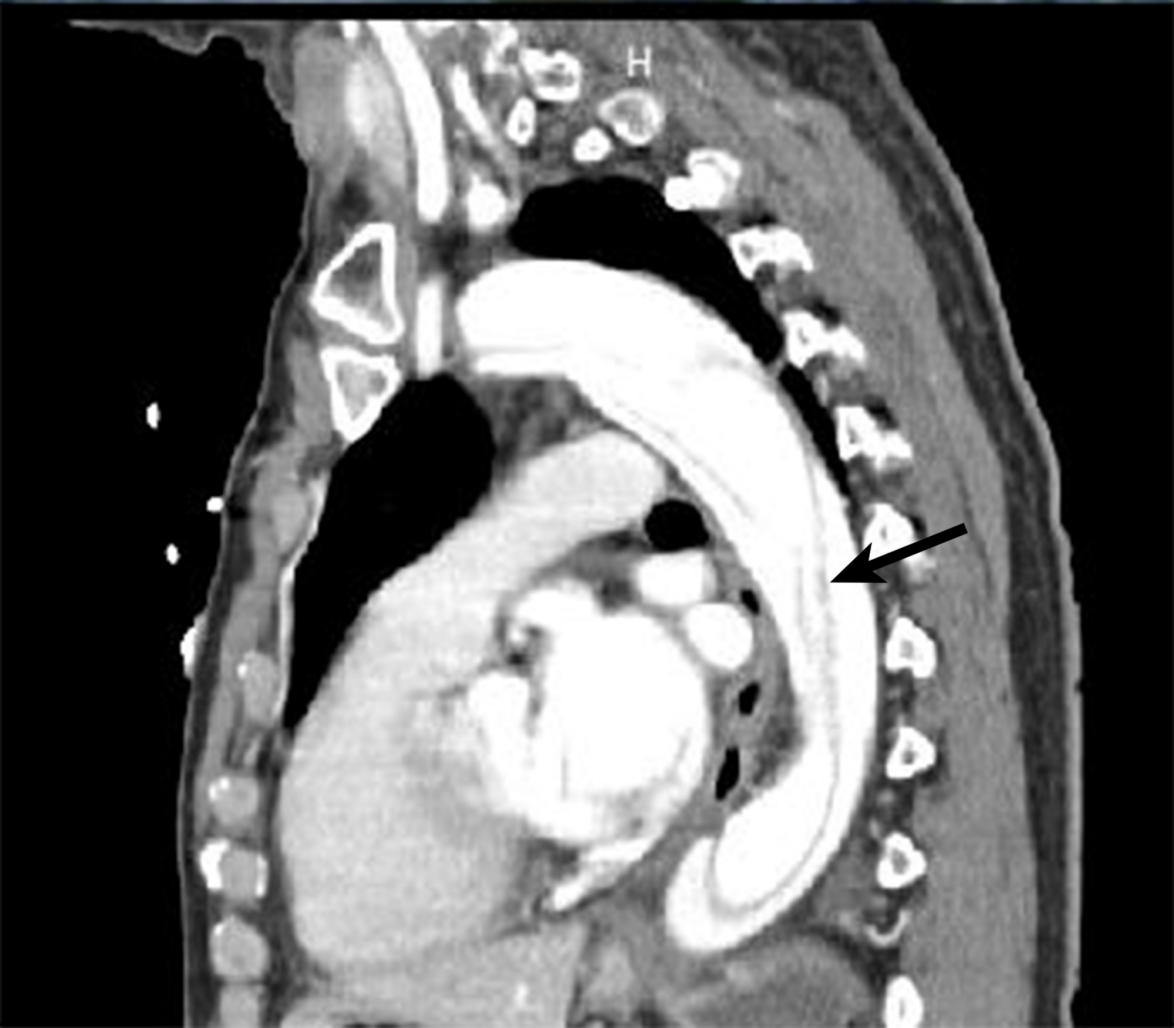
CTA showing type B AD with arch involvement

A CTA was performed and showed a type B AD with arch involvement beginning just distal to the takeoff of the left subclavian artery and extending to the infrarenal abdominal aorta (Figure 4).

The patient was started on an esmolol drip and admitted to the cardiothoracic surgery service.

An inpatient transthoracic echocardiogram (TTE) revealed a mild to moderate aneurysm of the ascending aorta. A repeat CTA on hospital day two showed no significant interval change of the AD. The patient was medically managed with anti-hypertensives and discharged on hospital day seven. Follow-up CTAs in three weeks, four months, and one year showed stable dissection.

## Discussion

AD is an uncommon diagnosis, with an estimated prevalence of 5,000 to 10,000 cases in the United States per year [4]. It is a difficult diagnosis for emergency physicians to make, and 15%-43% of cases are missed on initial presentation [4]. Missed diagnosis of AD is most common in patients with a working diagnosis of acute coronary syndrome, which is a much more prevalent condition [2]. The Stanford classification divides AD into type A, which involves the ascending aorta, and type B, which is limited to the aorta distal to the left subclavian artery. Type A AD is quoted to have a 1% per hour mortality and, even with surgical intervention, has a reported 30-day mortality of 17% [5]. Type B AD, typically managed medically, has a reported mortality of about 11%, and those on whom surgery is performed have a mortality rate of 31.5% [1]. CTA, magnetic resonance imaging (MRI), and transesophageal echocardiography (TEE) are all reliable diagnostic imaging modalities, reaching 100% sensitivity, and with CTA reaching 100% specificity [2].

Numerous case reports and series have demonstrated that POCUS performed by emergency physicians can diagnose abdominal and thoracic AD by the visualization of the dissection flap via the traditional transabdominal and transthoracic, specifically the parasternal long axis, views [6-9]. The transthoracic view has the disadvantage of visualizing only the proximal aorta and is thus not able to evaluate for type B AD. The suprasternal notch view, not part of routine cardiac POCUS, can be used to rapidly diagnose AD, primarily for type A AD. The view is obtained by placing the cardiac probe (2-4 MHz) over the sternal notch, indicator towards the head of the patient, and then slowly rotating the probe clockwise towards the patient’s left shoulder. One reference notes that this view is only useful for visualization of type A AD and not type B [3]. However, our case illustrates a clearly visualized type B AD with this view. One prior case report in the literature diagnosed a type B AD on suprasternal notch view in a similar manner [10]. As the innominate, left common carotid, and left subclavian arteries can be visualized in this view, either type A or B AD can be diagnosed [10].

## Conclusions

Cardiac POCUS can be used to rapidly diagnose AD if there is visualization of a dissection flap. While more commonly used to evaluate for type A AD, it is important to know that the suprasternal notch view may also be used to visualize a type B AD. The suprasternal notch view may provide imaging of the descending portion of the thoracic aorta, which can be missed by the cardiac parasternal long-axis view and the abdominal aorta view. In patients in whom there is suspicion for AD, the suprasternal notch view may be incorporated into a cardiac POCUS, as it may aid in the diagnosis of type B AD not visualized by other cardiac and aorta views.
